# The German Act on Autonomous Driving: Why Ethics Still Matters

**DOI:** 10.1007/s13347-022-00526-2

**Published:** 2022-04-04

**Authors:** Alexander Kriebitz, Raphael Max, Christoph Lütge

**Affiliations:** Chair of Business Ethics, TUM School of Governance, Technical University of Munich, Arcisstr. 21, 80333 Muenchen, Germany

**Keywords:** Autonomous driving, Ethics, Digital ethics, Artificial intelligence, Technical oversight

## Abstract

The German Act on Autonomous Driving constitutes the first national framework on level four autonomous vehicles and has received attention from policy makers, AI ethics scholars and legal experts in autonomous driving. Owing to Germany’s role as a global hub for car manufacturing, the following paper sheds light on the act’s position within the ethical discourse and how it reconfigures the balance between legislation and ethical frameworks. Specifically, in this paper, we highlight areas that need to be more worked out in the future either through ethical conventions, corporate measures or legal measures and examine how the law can be incorporated into the existing discourse on the regulation of technologies. Based on this examination, we derive implications for future discourse and elaborate on companies’ responsibilities in developing autonomous driving technologies in an ethical sense.

## Introduction

Recent technological progress in autonomous driving has given rise to a public debate on the required precautions for facilitating technological progress and on the ethical underpinnings for allowing autonomous driving. Public perception and media coverage have remained ambiguous about the consequences of autonomous driving, providing a mixed picture oscillating between scandalization and science fiction (Jelinski et al., 2021). Consequently, such entities as the United Nations Economic Commission for Europe (UNECE) (2020), sub-national legislation in California, Nevada and Arizona and ethics codes, as in the case of Germany, have sought to address societal concerns and formulate collective standards for autonomous driving to ensure trust in technologies integrated in automated or autonomous vehicles.^1^

This development has gained speed recently. In February 2021, Germany discussed a bill on autonomous driving, specifically addressing level four of autonomous driving, which entails a high level of automation. The act, which was finally passed in July 2021, marks an important step in autonomous driving legislation, as it depicts the first comprehensive national law on autonomous driving. Given Germany’s prowess in the car industry and the country’s aspirations to become a “world leader in autonomous driving,” the act will likely have a strong impact on international and EU regulations (BMVI, 2021). The evaluation of the act is therefore not only relevant for deciphering future tendencies of international standardization on autonomous driving but matters likewise for more general concerns, such as the long-term vision of sustainable traffic and practical questions about how to strike the right balance between voluntary ethics codes and binding legislation. Consequently, the contribution sheds light on the two following questions:

- How does the German law position itself in the existing discourse on the ethics of autonomous driving?
- What will be the future division between legislation and companies regarding enforcing and developing AI principles?

To answer both questions, the remainder of the paper is structured as follows: The first part concentrates on the partly overlapping discourses on the ethics of autonomous driving and of artificial intelligence (AI), under which autonomous driving is traditionally subsumed. The second part elaborates the act’s content with an explicit focus on ethics. In the subsequent discussion, we derive implications for the role of ethics discourse and examine the act’s implications for the future division of labor between legislation and ethics in autonomous driving. The paper specifically centers here on the observation that the act does not handle well the problem of unavoidable accidents and does not solve the problem that is rooted in the tension between principles of transparency and safety.

## Ethics of Artificial Intelligence and of Autonomous Driving

The 2010s witnessed the emergence of international, supranational and national frameworks and ethics codes that detail how to regulate autonomous systems making decisions independently from human beings. This development includes the regulation of autonomous driving in specific but also more general legal frameworks dedicated to AI. Given autonomous driving’s similarity and conceptual proximity to artificial intelligence, we will analyze both discourses to extrapolate the acts’ relative position within the ethics of technology discourse. First, we describe how the concepts of autonomous driving and artificial intelligence are related.

### Autonomous Driving as Part of Artificial Intelligence

To analyze the connection between the discourse on the ethics of artificial intelligence and on autonomous driving, it is important first to define both. The Merriam-Webster Dictionary (2021) defines AI as “the power of a machine to copy intelligent human behavior,” a definition that pertains to autonomous driving. The ethically relevant commonalities between autonomous driving and AI stem from to the fact that autonomous vehicles and AI are technologies that reduce human involvement, which might be prone to miscalculations or biases and which are based on data input (Kriebitz & Luetge, 2020). Moreover, AI makes decisions independently, rendering its path of decision making opaque ex ante, what explains why scholars often refer to AI as a black box (Rai, 2020). That said, AI solutions are increasingly used in autonomous vehicles equipped with multiple sensors collecting data and providing input for AI solutions that determine the vehicle’s path (Grigorescu et al., 2020). Autonomous driving is therefore subsumed within the category of AI because it raises comparable challenges and ethical issues.

### Ethics of Artificial Intelligence

As a matter of course, AI ethics inform the general question of how to deal with AI’s inherent and contingent properties. Recently, leading scholars in the field have specified several principles that aim to address AI’s various properties (Grigorescu et al., 2020). For instance, the AI4People framework (Floridi et al., 2018) defines five overarching principles for AI use and development, stating that AI should have a clear benefit (beneficence), be fair (fairness), not allow others to harm individuals (non-maleficence), be transparent and comprehensible for users (explicability) and not render human beings unfree (autonomy). Moreover, researchers have attached specific importance to biases due to data input or ill-calibrated algorithms that could lead to cases of discrimination against minorities (Floridi & Cowls, 2019; Max et al., 2021). Likewise, in human rights scholarship on AI, researchers have identified non-discrimination and human autonomy as the main pillars of ethical AI (Kriebitz & Luetge, 2020). These streams of literature have finally entered legislation in the proposed EU AI Act, the Singaporean Framework on AI and Smart Dubai. The commonality between these frameworks is the fact that they outline certain high-risk areas that require more human oversight and processes that mitigate adverse impacts on individual rights (Remolina & Saeh, 2019). “High risks” are identified when the consequences of AI are deemed irreversible or when an AI solution is likely to reproduce biases that affect vulnerable or historically disadvantaged groups negatively.

### Ethics of Autonomous Driving

The specific role of autonomous driving within the AI ethics discourse stems from the fact that mistakes and miscalculations could have sizable effects on individuals’ lives, limbs and property (Luetge, 2017). Indeed, lethal accidents have received intensive media coverage, forcing ethicists, policy wonks and legal experts to contemplate the creation of specific frameworks concentrating on autonomous driving (Favarò et al., 2017). Relevant frameworks of autonomous driving distinguish here between different automation levels (Graphic 1).

**Graphic 1:**
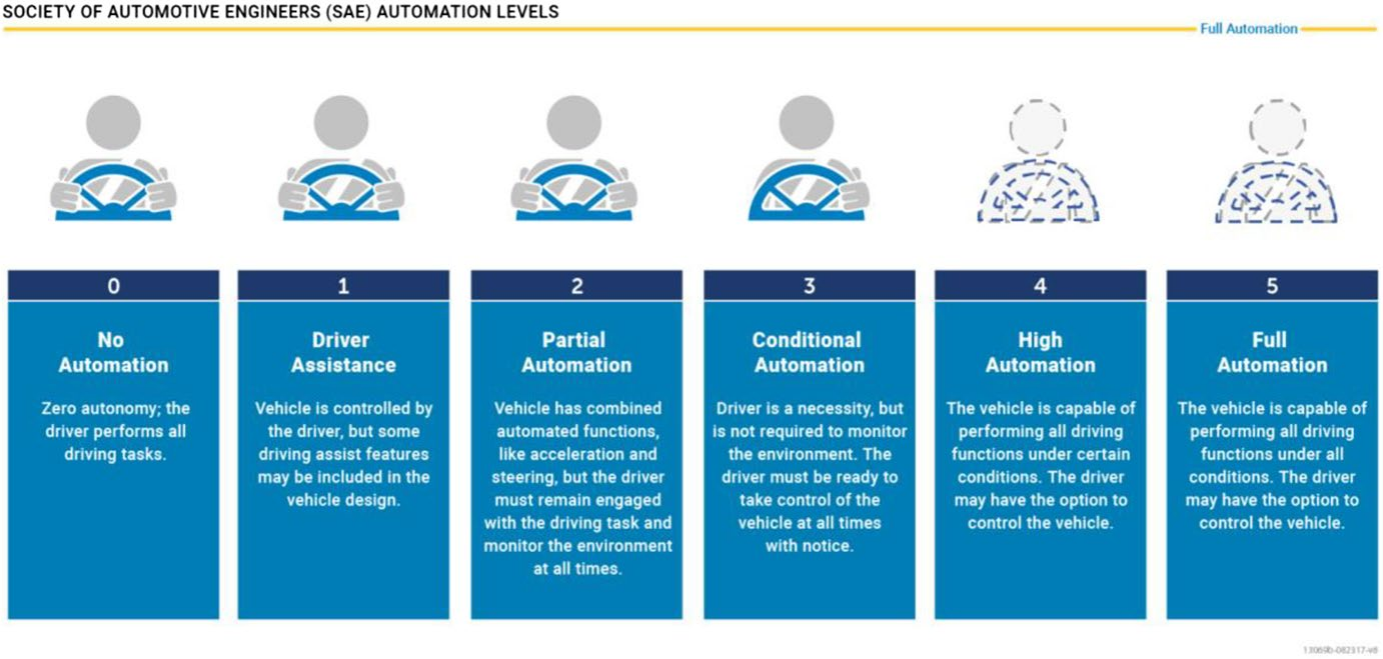
Levels of Autonomous Driving. Courtesy: NHTSA

Conceptually, the various levels of autonomous driving correspond not so much to the level of technical sophistication but rather to the degree of driver involvement and autonomy. The concept of various levels of autonomous driving therefore harmonizes with the risk classification provided by AI frameworks (Data Ethics Commission of the Federal Government, 2019). Given the strong role of AI solutions at *level four* and *level five*, the ethics discourse has accompanied the development of this technology early on by pointing out the ethical advantages of autonomous driving in terms of safety, comfort and sustainability, formulating concerns and highlighting risks. Although earlier research has demonstrated that most traffic accidents are the result of human miscalculations, autonomous driving is affected by a different set of safety issues, including hacking attacks or technical failure. These non-traditional safety issues have hence emerged as a new field of research. Apart from safety questions and other practical issues, unavoidable crash situations are a controversially discussed aspect of autonomous driving. These situations might occur when a car’s AI mechanism has to decide whether to hit an elderly woman or a young child, other options being unavailable. Given the major ramifications of such hypothetical dilemmas on non-discrimination, dignity and human rights, unavoidable crash situations have been widely discussed in ethics, philosophy and experimental research (cf. Gogoll and Müller, 2017; Awad et al., 2018). Researchers have recently stressed the fact that the ethics of autonomous driving involve the management of risks (Bonnefon et al., 2019). This fact applies specifically to trajectory planning. The programming method could create greater risks in various traffic situations when determining the autonomous vehicle’s correct distance from other close objects, such as cyclists or trucks (Geisslinger et al., 2021).

Confronted with these autonomous-driving-specific challenges, international efforts have concentrated on autonomous driving, most prominently those of the UNECE and the International Telecommunication Union (ITU). The UNECE has created several principles of autonomous driving, which include, for example, that “an automated/autonomous vehicle shall not cause any non-tolerable risk” (UNECE, 2020, p. 2). A further notable step for incorporating and reflecting the ethical discourse in terms of standardization has been the German Ethics Commission on Autonomous Driving (2017), involving former judges of the constitutional court, experts, ethicists and representatives of religious denominations (cf. Luetge, 2017). Specific questions about the general AI discourse have been discussed in the guidelines, including the question of unavoidable crash situations, in which the setting off of lives was deemed illegitimate to distinguish by personal features in dilemmas, broader safety issues, questions pertaining to data use and the elaboration of the responsibilities of owners, drivers and producers of autonomous vehicles. In the following section, we refer to the final report of the German Ethics Commission on Autonomous Driving as the “German Ethics Code.” The code largely reflects the German Constitutional Court’s positions on dilemmas involving normative questions, such as human dignity, non-discrimination and equality before the law.

## The Act and Its Legal Consequences

Given the multiple ethical questions about autonomous driving, the act, which was passed in July 2021, is characterized by the quest to differentiate between various essential norms and therefore relates to constitutionally enshrined rights, such as human dignity, the right to life and the right to non-discrimination. Likewise, the right introduces a technology that is both new and complex. For this purpose, the act addresses the very definition of autonomous driving, general division of responsibilities and assignment of tasks as mandated by legislators and precautions taken to prevent accidents. As the act covers various implications for insurances and other procedural aspects, the following summary will largely center on the ethically relevant questions stated in the preceding literature review.

### Definition of Autonomous Driving

First and foremost, the act establishes a legal framework for allowing and realizing autonomous driving in designated areas. The majority of the regulation appears to concern public and not private transportation given the high costs of maintenance and complex legal questions that are revealed in the section on the bill’s costs.^2^ A further notable aspect of the law is that it explicitly addresses higher stages of autonomous driving, namely *level four,* as level three, a mixed type between human involvement and autonomous driving, has been covered by an earlier amendment to the Road Traffic Act, which defines an autonomously driving vehicle primarily as one that.

*“die Fahraufgabe ohne eine fahrzeugführende Person selbstständig in einem festgelegten Betriebsbereich erfüllen kann.” - “can perform the driving task independently within a specified operating range without a person driving the vehicle.”*^3^

Moreover, the act elaborates on the technical requirements of autonomous vehicles, including a software system that can operate without permanent supervision of the technical oversight or driver, contains an accident mitigation and reduction system and can initiate a “minimal-risk state.”

### General Division of Responsibilities

To understand the law’s multiple implications, it is necessary to detail the interplay between the actors involved in autonomous driving. The act distinguishes between roles that are relevant to comprehend the technology’s design and introduces the novel category of “technical oversight.” According to the act, technical oversight comes from a natural person who can deactivate the autonomous vehicle and approve certain maneuvers of the car. The design proposed in the act resembles a remote control function with a kill switch function. This kill switch function has also been discussed in earlier regulations. For example, the UNECE refers to a “fail safe response.” However, the meaning of both concepts varies, as the German approach is more about remote control than automated processes.^4^ Consequently, the law requires a stable connection between autonomous vehicles and technical oversight that allows for supervision. Likewise, the car’s owner and producer have significant responsibilities, the prior for regular updates as well as maintenance and the latter for risk assessments, technical compatibility and training.^5^ It is noteworthy that producers are responsible for detecting manipulation and hacking attacks, a topic that has made headlines recently (Petit & Shladover, 2014).

### Precautions to Mitigate and Prevent Accidents

When elaborating on the precautions to reduce fatalities, the act introduces a new legal term in German legislation, which is called “risk-minimized state.” The law explicitly defines this state as follows:

*“Risikominimaler Zustand im Sinne dieses Gesetzes ist ein Zustand, in dem sich das Kraftfahrzeug mit autonomer Fahrfunktion auf eigene Veranlassung oder auf Veranlassung der Technischen Aufsicht an einer möglichst sicheren Stelle in den Stillstand versetzt und die Warnblinkanlage aktiviert, um unter angemessener Beachtung der Verkehrssituation die größtmögliche Sicherheit für die Fahrzeuginsassen, andere Verkehrsteilnehmerin und Dritte zu gewährleisten.”*

*“For the purposes of this Act, a risk-minimized state is a state in which the motor vehicle with autonomous driving function, at its own instigation or at the instigation of the technical supervisor, comes to a standstill in the safest possible place and activates the hazard warning lights in order to ensure the greatest possible safety for the vehicle occupants, other road users and third parties, taking due account of the traffic situation.”*

The risk-minimized state describes a maneuver that allows the car to stop safely in the case of a technical interruption to protect passengers’ and other parties’ lives and limbs and plays a major role in accident mitigation as described in the law and as mandated in the German Ethics Code.

Furthermore, the act details the general setup of ethical requirements for autonomous driving by establishing three guiding principles that establish the key norms of autonomous driving. As a general rule, the system should aim to reduce road fatalities and, more important, prioritize human life over other considerations, such as potential damage done to property. This effort corresponds to the risk-minimized condition the UNECE (2020) mentioned and to earlier remarks from the ethics commission on prioritizing human life over animals and property.^6^ Consequently, the act demands an accident prevention and mitigation system that.

*“für den Fall einer unvermeidbaren alternativen Schädigung unterschiedlicher Rechtsgüter die Bedeutung unterschiedlicher Rechtsgüter berücksichtigt, wobei der Schutz menschlichen Lebens die höchste Priorität besitzt”*

*“in the case of unavoidable alternative harm to different legal interests, takes into account the importance of different legal interests, with the protection of human life having the highest priority.”*

The third rule is that unavoidable crashes should not involve discrimination based on personal features, as the act requires a system that.

*“Für den Fall einer unvermeidbaren alternativen Gefährdung von Menschenleben keine weitere Gewichtung Anhang persönlicher Merkmale vorsieht.*

*“in the event of an unavoidable alternative risk to human life does not factor in personal characteristics.”*

Further requirements focus on technical propositions, including proposals for new maneuvers after the risk-minimized state, measures to counteract maneuvers initiated by the technical supervisor that would threaten other traffic participants, mechanisms to raise awareness of malfunctions, functions to detect barriers and limitations of the technical system and that subsequently initiate the risk-minimized state, tools for the technical oversight to deactivate autonomous driving and a connection that is protected against unauthorized access.

### Data Exchange

The next section is devoted to the regulation of data exchange. The relationship between owner, technical oversight and producer as well as the constant connection between technical oversight and the device involves not only a huge amount of data storage in the form of a black box but also data exchange, which is needed to improve autonomous vehicles’ capacities and detect flaws and deficits. This exchange concerns data, such as geolocation, use times, alternative driving maneuvers, environmental conditions, speed and communication, which the German Federal Motor Transport Authority can request (Kraftfahrt-Bundesamt). Such a request concerns, in particular, the provision of data to research accidents. The exchange of anonymous data would involve research facilities and universities that are researching autonomous driving.

The remainder of the act is then devoted to procedural questions, exemptions from the law and insurance-related questions.

## Discussion

The act entails several implications for general tendencies in the ethics of autonomous driving and for corporate responsibilities to ensure ethical conduct in the development of autonomous driving solutions.

### The Act’s Role in the Ethics discourse

The act has multiple implications for ethics discourse. The most important one is that the law needs to be seen in the context of legislation on consumer protection, product safety, non-discrimination and data protection and is an insufficient normative source on its own to fully gauge autonomous driving’s ethical implications.

#### Proof of Concept Design

The first observation is that the law does not center so much on the private sector but rather on corporate and public use. Moreover, the technology requires a stable cellular connection, which excludes the broad use of autonomous cars in remote areas. The law will therefore require major changes to make it compatible with individual and private traffic and is therefore not final legislation for autonomous driving in its entirety but a first and cautious move to allow the technology to be used in specifically designated areas and by fewer users. The law therefore can rather be regarded as a proof of concept that will later be amended or changed.

#### Integration of Earlier Ethical Concerns

From an ethical perspective, the integrative approach of minding ethics and human values while focusing on an open approach to technology is nonetheless an important precondition for later legislation. It stands clearly in the tradition of the report of the ethics commission, specifically regarding ways to prevent “unavoidable” scenarios. This idea is expressed in the conception of the risk-minimized position, the act’s overall mission statement to reduce and prevent accidents and so forth. Moreover, the bill preceding the act refers to the ethical benefits of using autonomous cars, expressed as safety gains, comfortability and sustainability.

#### Data Ethics

The act’s design suggests that the exchange of data will be massive given the concept of technical oversight. However, the law needs to be interpreted by integrating already existing frameworks at the European and national levels, especially in already existing legislation on data protection, such as the GDPR. The integration of the explainability principle from AI ethics mandating that users should know the amount and type of data transmitted to other parties is very valuable here; however, the practical realization of this principle will depend on best practices by companies and future research.

#### Open Questions

The proof of concept design explains why many questions remain open. Although the act includes general principles for unavoidable situations, the suggestions are less detailed than in the German Ethics Code, which had repeatedly dealt with unavoidable crash situations. The passage “*in the event of an unavoidable alternative risk to human life does not factor in personal characteristics*” does not illuminate what personal characteristics are, nor does it state whether autonomous vehicles might hit individuals violating traffic rules in unavoidable accidents. These questions have been posed in the Moral Machine experiment, igniting a moral debate on the nature of discrimination in the context of autonomous driving. A possible scenario would be an autonomous vehicle having to decide between hitting a jaywalker or a random person obeying the law. Whether the obedience to law in a given situation is a personal characteristic comparable to ethnicity, body size and shape, age, gender, etc., is questionable. Likewise, the law addresses whether maneuvers in unavoidable crashes should be based on the analysis of the potential number of persons hit. It appears plausible that the act follows a Kantian perspective on this matter, given the view espoused by the ethics commission and rulings of the German Constitutional Court against offsetting human lives in dilemmas. Nevertheless, a clear statement would be needed to navigate moral dilemmas from the developers’ and users’ perspectives. Moreover, this question will require a solution and should not be left to companies to decide but rather the public sector as the German Ethics Code suggests because companies lack the moral legitimacy to engage in such derivations from fundamental norms when ethics do not provide unambiguous answers.^7^

### Companies’ Responsibilities

The gaps that the law leaves unaddressed will need to be addressed by various actors, most importantly by the legislator and jurisprudence that will not only consider the act as a sole normative source but also refer to constitutional norms and other legal sources that bear implications for autonomous driving, including data protection legislation at the EU level, non-discrimination frameworks and AI-specific legislation presented in the EU AI Act’s proposal. Nevertheless, companies and developers of autonomous driving solutions will continue to play a significant role in implementing the framework and creating best practice solutions. In general, the authors express the view that two areas will be critical for autonomous driving.

#### Transparency

COVID-19 has revealed that trust in and transparency of institutions plays a significant role in the acceptance of new technologies and legislation. Consequently, companies will have to demonstrate the active and passive safety of AVs and report on the safety impact of autonomous driving. This effort might include the following questions: How do autonomous vehicles perform in terms of safety? What accident patterns emerge? Some companies have already published some information on these issues, but more input will be needed from neutral and accepted information sources. Companies’ audited sustainability reports are a likely entry for disclosure of more information, as product safety is already reflected in CSR legislation and as CSR legislation is gaining more prominence due to new EU legislation.

#### Cybersecurity

Transparency, however, comes with certain limitations: Producers and providers of autonomous vehicles will also have to consider the question of cybersecurity, as envisioned in the German Ethics Code.^8^ Transparency might be a double-edged sword, as open source intelligence (OSINT) is an often underestimated factor in successful hacking attacks (Ball et al., 2012). Cyberattacks and hacking by terrorists relying on OSINT could cause lethal damage and result in irreversible consequences (Lee & Lim, 2016). Moreover, autonomous vehicles could be used as part of botnets and cause sizable damage to third parties. As a result, companies will face strong moral pressure to invest in a reliable cyber defense architecture and constantly search for multiple entry points for potential cyberattacks.

#### Design of Human–Machine Interfaces

Ethics, however, do not only play an important role as a set of normative principles but also in their experimental form to identify the right design for human–machine interfaces and as a means to identify human beings’ specific biases and behaviors. Integrating these findings is therefore a major aspect of the development of AI and autonomous driving solutions. This area is particularly relevant for level three in autonomous driving, which has been covered by an amendment of the German Road Traffic Act and the German Ethics Code. Key requirements include a clear distinction for the driver between autonomous and conventional driving.^9^ Moreover, manufacturers of level three vehicles have to create functions allowing the driver to override the AI and establish a clear distinction between autonomous and human driving.

In a nutshell, the task of standardizing approaches to autonomous driving can hardly be done by legislators alone but requires a multi-stakeholder exchange involving consumer protection watchdogs, cybersecurity experts, governmental institutions and car manufacturers. As individual entities, companies play a decisive role in establishing ethics of autonomous driving, Germany’s new Act on Autonomous Driving notwithstanding.

## Concluding Remarks

Germany’s act has paved the way to legislating higher levels of autonomous driving while international initiatives have been stalling. Moreover, the act sets the stage to create a legal framework for autonomous driving at higher levels, indicating a potential shift of priorities toward higher levels of autonomy before implementing hybrid types, blurring responsibilities between humans and machines.

The act can therefore be considered a decisive move in the AI ethics discourse and the autonomous driving discourse, enabling companies to develop and test autonomous vehicles. However, several details of autonomous driving remain to be worked out either by later amendments and changes to the law or by jurisprudence. This effort includes primarily the specification of the term “personal properties” in dilemmas as well as the interpretation of non-discrimination in connection with unavoidable crash situations. Given the law’s focus on the public sector in very limited areas, such cases are so far not urgent and therefore less likely. However, the expansion of autonomous driving in the private sector will render legal clarification of this type more important.

Another takeaway is that ethics need to be integrated into autonomous driving solutions and be part of the development early on. This development represents an integrative instrument that looks at autonomous driving through the lens of stakeholder expectations expressed in consumer rights, non-discrimination, fairness, inclusion and human dignity. Given this important role of ethics from the beginning, ethics will play a lasting role in the discourse on autonomous driving. This discourse applies in specifically to companies that are largely responsible for the design of human–machine interfaces, explainability of AI solutions and cybersecurity.

## Footnotes

1 US Senate Bill No. 1298, Chapter 570; https://leginfo.legislature.ca.gov/faces/billNavClient.xhtml?bill_id=201120120SB1298

2 Bill “Entwurf eines Gesetzes zur Änderung des Straßenverkehrsgesetzes und des Pflichtversicherungsgesetzes – Gesetz zum autonomen Fahren” (08.02.2021) https://www.bmvi.de/SharedDocs/DE/Anlage/Gesetze/Gesetze-19/gesetz-aenderung-strassenverkehrsgesetz-pflichtversicherungsgesetz-autonomes-fahren.pdf?__blob=publicationFile

3 Compare: Gesetz zur Änderung des Straßenverkehrsgesetzes und des Pflichtversicherungsgesetzes—Gesetz zum autonomen Fahren. [Act on Autonomous Driving] § 1d(1)1.

4 The automated process corresponds rather to the minimum-risk maneuver that is described in the Section 2.3.

5 Compare: Gesetz zur Änderung des Straßenverkehrsgesetzes und des Pflichtversicherungsgesetzes—Gesetz zum autonomen Fahren. [Act on Autonomous Driving] § 1f.

6 Compare Article 7 of the Ethics Code: “Thus, within the constraints of what is technologically feasible, the systems must be programmed to accept damage to animals or property in a conflict if this means that personal injury can be prevented.”.

7 Art. 8: *“For this reason, perhaps more than any other, it would be desirable for an independent public sector agency (for in- stance a Federal Bureau for the Investigation of Accidents Involving Automated Transport Systems or a Federal Office for Safety in Automated and Connected Transport) to systematically process the lessons learned.”* Moreover, we express the view that companies’ legislation for such preferences would be unconstitutional.

8 Automated driving is justifiable only to the extent to which conceivable attacks, in particular manipulation of the IT system or innate system weaknesses, do not result in such harm as to lastingly shatter people’s confidence in road transport.

9 Compare Art.16: It must be possible to determine clearly whether a driverless system is being used or a driver retains accountability with the option of overruling the system.
